# Supplementary material for: Detailed molecular and epigenetic characterization of the pig IPEC-J2 and chicken SL-29 cell lines
Source: iScience. 2023 Feb 20;26(3):106252. doi: 10.1016/j.isci.2023.106252 (PMC10018572; doi:10.1016/j.isci.2023.106252)
Supplement: Data S2. Complete homer output for identified motifs in Chicken SL-29, related to Tables 5 and 6 — Homer motif analysis results for histone modifications H3K4me1, H3K4me3, H3K27ac, enhancers, and ATAC-seq of chicken SL-29 cell line. Parameters for possible false positives is as mentioned earlier for S5. [file mmc3.zip › Data_S2/S6/Chicken_SL_29/peak_files_H3K4me3/knownResults.html]

peak\_files\_H3K4me3 - Homer Known Motif Enrichment Results


# Homer Known Motif Enrichment Results (peak\_files\_H3K4me3)

Homer *de novo* Motif Results  
Gene Ontology Enrichment Results  
Known Motif Enrichment Results (txt file)  
Total Target Sequences = 17176, Total Background Sequences = 32696

|  |  |  |  |  |  |  |  |  |  |  |  |
| --- | --- | --- | --- | --- | --- | --- | --- | --- | --- | --- | --- |
| Rank | Motif | Name | P-value | log P-pvalue | q-value (Benjamini) | # Target Sequences with Motif | % of Targets Sequences with Motif | # Background Sequences with Motif | % of Background Sequences with Motif | Motif File | SVG |
| 1 | C T A G T C G A C T G A C G T A T A C G G A C T T C A G T C G A G T C A T G C A T A C G A G C T | IRF2(IRF)/Erythroblas-IRF2-ChIP-Seq(GSE36985)/Homer | 1e-183 | -4.216e+02 | 0.0000 | 675.0 | 3.93% | 333.5 | 1.02% | motif file (matrix) | svg |
| 2 | T C A G C T G A C G T A C G T A T A C G G C A T C T A G C T G A C G T A C G T A T A C G G A C T | IRF1(IRF)/PBMC-IRF1-ChIP-Seq(GSE43036)/Homer | 1e-171 | -3.959e+02 | 0.0000 | 734.0 | 4.28% | 407.6 | 1.25% | motif file (matrix) | svg |
| 3 | C T G A A T C G A G C T A G C T A C G T T A G C C T G A T A C G C G A T A C G T G A C T A G T C | ISRE(IRF)/ThioMac-LPS-Expression(GSE23622)/Homer | 1e-159 | -3.680e+02 | 0.0000 | 420.0 | 2.45% | 150.9 | 0.46% | motif file (matrix) | svg |
| 4 | C T G A T A C G G C A T A G C T A G C T A G T C T C G A A C T G C A G T A G C T A G C T G A T C | IRF3(IRF)/BMDM-Irf3-ChIP-Seq(GSE67343)/Homer | 1e-122 | -2.822e+02 | 0.0000 | 1098.0 | 6.40% | 947.5 | 2.91% | motif file (matrix) | svg |
| 5 | T C A G T C A G G C T A C G T A T A C G G A C T T C A G T C G A C T G A C G T A T A C G G A C T | IRF8(IRF)/BMDM-IRF8-ChIP-Seq(GSE77884)/Homer | 1e-121 | -2.801e+02 | 0.0000 | 1141.0 | 6.65% | 1005.8 | 3.08% | motif file (matrix) | svg |
| 6 | C T A G C T A G C G T A C G T A T A C G C G A T C T A G C T G A C T G A C G T A T A C G G A C T | PU.1:IRF8(ETS:IRF)/pDC-Irf8-ChIP-Seq(GSE66899)/Homer | 1e-61 | -1.422e+02 | 0.0000 | 591.0 | 3.44% | 523.2 | 1.60% | motif file (matrix) | svg |
| 7 | T C G A T G A C G C A T A G C T C A G T G A T C G C T A G A T C G A C T A C G T G C A T A G T C | PRDM1(Zf)/Hela-PRDM1-ChIP-Seq(GSE31477)/Homer | 1e-49 | -1.136e+02 | 0.0000 | 1349.0 | 7.86% | 1685.9 | 5.17% | motif file (matrix) | svg |
| 8 | G C T A T A G C A G C T A T C G G T C A C G T A G C T A A T G C G A T C C T G A | IRF4(IRF)/GM12878-IRF4-ChIP-Seq(GSE32465)/Homer | 1e-43 | -1.010e+02 | 0.0000 | 1130.0 | 6.58% | 1390.4 | 4.26% | motif file (matrix) | svg |
| 9 | T G C A A C T G T A C G A T G C A G T C G A C T T C G A A T C G | ZNF711(Zf)/SHSY5Y-ZNF711-ChIP-Seq(GSE20673)/Homer | 1e-31 | -7.351e+01 | 0.0000 | 5053.0 | 29.43% | 8293.7 | 25.43% | motif file (matrix) | svg |
| 10 | A G T C C T G A A T C G A G C T A G C T G A C T A G T C G C T A A C G T C G A T G C A T C G A T A T C G C G T A T A G C G C A T A T G C C G T A | bZIP:IRF(bZIP,IRF)/Th17-BatF-ChIP-Seq(GSE39756)/Homer | 1e-30 | -6.915e+01 | 0.0000 | 939.0 | 5.47% | 1207.7 | 3.70% | motif file (matrix) | svg |
| 11 | T G A C C T A G T C A G G T C A C G T A T C A G C G A T T C A G T C G A T G C A C T G A T A G C | PU.1-IRF(ETS:IRF)/Bcell-PU.1-ChIP-Seq(GSE21512)/Homer | 1e-26 | -6.166e+01 | 0.0000 | 2942.0 | 17.14% | 4624.2 | 14.18% | motif file (matrix) | svg |
| 12 | C T G A A C T G A C T G A G T C A G T C A G C T C T A G T A C G | ZFX(Zf)/mES-Zfx-ChIP-Seq(GSE11431)/Homer | 1e-25 | -5.962e+01 | 0.0000 | 2680.0 | 15.61% | 4181.3 | 12.82% | motif file (matrix) | svg |
| 13 | C T G A T C A G G T A C G C T A A C T G T G A C G C A T C A T G | SCL(bHLH)/HPC7-Scl-ChIP-Seq(GSE13511)/Homer | 1e-24 | -5.694e+01 | 0.0000 | 10698.0 | 62.31% | 19055.7 | 58.44% | motif file (matrix) | svg |
| 14 | A T C G A G T C A G T C G A C T A T G C C T G A C T A G A C T G T A C G G T A C C T G A C G A T | AP-2gamma(AP2)/MCF7-TFAP2C-ChIP-Seq(GSE21234)/Homer | 1e-19 | -4.499e+01 | 0.0000 | 4617.0 | 26.89% | 7785.9 | 23.88% | motif file (matrix) | svg |
| 15 | A T G C G A C T A G C T A G C T A G T C G C T A C A G T C G A T G C T A A C G T A C T G G C T A T A G C G C A T T G A C | IRF:BATF(IRF:bZIP)/pDC-Irf8-ChIP-Seq(GSE66899)/Homer | 1e-19 | -4.469e+01 | 0.0000 | 310.0 | 1.81% | 335.8 | 1.03% | motif file (matrix) | svg |
| 16 | G C T A C G A T C A T G A T G C A G T C A G T C G A C T T A C G T C G A C T A G A C T G T A G C | AP-2alpha(AP2)/Hela-AP2alpha-ChIP-Seq(GSE31477)/Homer | 1e-18 | -4.310e+01 | 0.0000 | 3618.0 | 21.07% | 5994.7 | 18.38% | motif file (matrix) | svg |
| 17 | G A C T T C G A C G A T T A C G C T A G T A C G A C T G A T G C G T A C G T A C | Zac1(Zf)/Neuro2A-Plagl1-ChIP-Seq(GSE75942)/Homer | 1e-14 | -3.451e+01 | 0.0000 | 6858.0 | 39.95% | 12068.0 | 37.01% | motif file (matrix) | svg |
| 18 | C A G T T G C A A C G T A C T G C G T A A T C G C G A T T G A C C G T A A C G T | BATF(bZIP)/Th17-BATF-ChIP-Seq(GSE39756)/Homer | 1e-12 | -2.799e+01 | 0.0000 | 933.0 | 5.43% | 1401.0 | 4.30% | motif file (matrix) | svg |
| 19 | T A C G G A C T A C T G A C T G C T A G A T G C A G T C A G T C A G T C C T G A | ZNF692(Zf)/HEK293-ZNF692.GFP-ChIP-Seq(GSE58341)/Homer | 1e-11 | -2.689e+01 | 0.0000 | 425.0 | 2.48% | 567.7 | 1.74% | motif file (matrix) | svg |
| 20 | C T A G T C G A A C G T A C T G C G T A A T G C A C G T G T A C C G T A A G C T G A T C G T A C | Atf3(bZIP)/GBM-ATF3-ChIP-Seq(GSE33912)/Homer | 1e-11 | -2.589e+01 | 0.0000 | 965.0 | 5.62% | 1469.8 | 4.51% | motif file (matrix) | svg |
| 21 | C A T G A G T C C T G A T G A C A T C G G A C T G T C A A G T C T A G C G A T C | HIF2a(bHLH)/785\_O-HIF2a-ChIP-Seq(GSE34871)/Homer | 1e-10 | -2.471e+01 | 0.0000 | 1421.0 | 8.28% | 2268.7 | 6.96% | motif file (matrix) | svg |
| 22 | T C G A A C G T C A T G G C T A T A G C C G A T G T A C G C T A A C G T A T G C | AP-1(bZIP)/ThioMac-PU.1-ChIP-Seq(GSE21512)/Homer | 1e-10 | -2.390e+01 | 0.0000 | 1053.0 | 6.13% | 1635.1 | 5.01% | motif file (matrix) | svg |
| 23 | C T A G T C G A G C A T C A T G G C T A T A G C C G A T G T A C C T G A A G C T | JunB(bZIP)/DendriticCells-Junb-ChIP-Seq(GSE36099)/Homer | 1e-9 | -2.280e+01 | 0.0000 | 783.0 | 4.56% | 1181.2 | 3.62% | motif file (matrix) | svg |
| 24 | A C T G C T A G T C G A C G A T C A T G G C T A A T C G C G A T G T A C G C T A A G C T G T A C | Fra1(bZIP)/BT549-Fra1-ChIP-Seq(GSE46166)/Homer | 1e-9 | -2.092e+01 | 0.0000 | 808.0 | 4.71% | 1237.2 | 3.79% | motif file (matrix) | svg |
| 25 | A C T G C A T G T C G A A C G T A C T G C G T A A T C G A C G T G T A C C G T A G A C T A G T C | Fos(bZIP)/TSC-Fos-ChIP-Seq(GSE110950)/Homer | 1e-8 | -2.066e+01 | 0.0000 | 850.0 | 4.95% | 1312.0 | 4.02% | motif file (matrix) | svg |
| 26 | T G C A A T G C A C G T A C G T A C G T A T G C C T A G A C G T A C G T A G C T G A T C A G C T | T1ISRE(IRF)/ThioMac-Ifnb-Expression/Homer | 1e-8 | -1.968e+01 | 0.0000 | 48.0 | 0.28% | 34.1 | 0.10% | motif file (matrix) | svg |
| 27 | C A T G C T A G T C G A A C G T A C T G C G T A T A G C C G A T T G A C C G T A A G C T G A T C | Fra2(bZIP)/Striatum-Fra2-ChIP-Seq(GSE43429)/Homer | 1e-8 | -1.904e+01 | 0.0000 | 671.0 | 3.91% | 1017.4 | 3.12% | motif file (matrix) | svg |
| 28 | T C G A T G C A A C T G G T C A A G T C A G T C A G T C A G T C A G C T T G A C | LRF(Zf)/Erythroblasts-ZBTB7A-ChIP-Seq(GSE74977)/Homer | 1e-7 | -1.744e+01 | 0.0000 | 5281.0 | 30.76% | 9413.8 | 28.87% | motif file (matrix) | svg |
| 29 | A T G C T C G A T A C G A C G T A T G C A G T C A C G T A G T C A G T C G A T C | Znf263(Zf)/K562-Znf263-ChIP-Seq(GSE31477)/Homer | 1e-7 | -1.680e+01 | 0.0000 | 6181.0 | 36.00% | 11109.5 | 34.07% | motif file (matrix) | svg |
| 30 | T A G C C G T A C T G A T A C G C G T A A C G T A C T G A C T G A G T C T A C G C T A G G T A C | YY1(Zf)/Promoter/Homer | 1e-7 | -1.634e+01 | 0.0000 | 423.0 | 2.46% | 617.9 | 1.89% | motif file (matrix) | svg |
| 31 | T C G A T C G A T A C G G A T C G T C A G T A C C G A T A G C T G T C A T G C A | Nkx3.1(Homeobox)/LNCaP-Nkx3.1-ChIP-Seq(GSE28264)/Homer | 1e-6 | -1.483e+01 | 0.0000 | 4242.0 | 24.71% | 7533.8 | 23.10% | motif file (matrix) | svg |
| 32 | T C A G G C T A T C A G C A G T T G A C G T C A A G T C A T C G T G C A G T A C C A G T G A T C | Npas4(bHLH)/Neuron-Npas4-ChIP-Seq(GSE127793)/Homer | 1e-6 | -1.476e+01 | 0.0000 | 2848.0 | 16.59% | 4962.8 | 15.22% | motif file (matrix) | svg |
| 33 | T A C G T A C G G T A C A T C G A C T G T A C G T C G A C T G A T C G A A T C G | E2F6(E2F)/Hela-E2F6-ChIP-Seq(GSE31477)/Homer | 1e-6 | -1.400e+01 | 0.0000 | 3538.0 | 20.61% | 6246.6 | 19.16% | motif file (matrix) | svg |
| 34 | C T A G T C G A A C G T A C T G C G T A T A G C C G A T G T A C C G T A A G C T G A T C G T A C | Jun-AP1(bZIP)/K562-cJun-ChIP-Seq(GSE31477)/Homer | 1e-5 | -1.336e+01 | 0.0000 | 336.0 | 1.96% | 490.5 | 1.50% | motif file (matrix) | svg |
| 35 | T A G C G C A T A G T C G A T C A T G C G A C T C T A G A C T G A C T G C T G A A C T G C T A G A G T C T G A C C G A T | GLIS3(Zf)/Thyroid-Glis3.GFP-ChIP-Seq(GSE103297)/Homer | 1e-5 | -1.305e+01 | 0.0000 | 5309.0 | 30.92% | 9560.6 | 29.32% | motif file (matrix) | svg |
| 36 | A G T C G C A T C G T A C G T A G T A C A C G T A C T G G A T C G A T C T C G A | BMYB(HTH)/Hela-BMYB-ChIP-Seq(GSE27030)/Homer | 1e-5 | -1.301e+01 | 0.0000 | 3616.0 | 21.06% | 6410.0 | 19.66% | motif file (matrix) | svg |
| 37 | C T A G C T A G A G T C T C A G A C T G A C G T A C G T C T G A | MYB(HTH)/ERMYB-Myb-ChIPSeq(GSE22095)/Homer | 1e-5 | -1.282e+01 | 0.0000 | 4708.0 | 27.42% | 8443.8 | 25.89% | motif file (matrix) | svg |
| 38 | C T A G T C G A C G A T A C T G C G T A T A C G A G C T T G A C G C T A A C G T G A T C T A G C | Fosl2(bZIP)/3T3L1-Fosl2-ChIP-Seq(GSE56872)/Homer | 1e-4 | -1.110e+01 | 0.0002 | 445.0 | 2.59% | 690.2 | 2.12% | motif file (matrix) | svg |
| 39 | A C T G A C G T C A T G A T C G A T C G T G A C A C T G A T C G A T C G T G C A C T G A C G T A | E2F3(E2F)/MEF-E2F3-ChIP-Seq(GSE71376)/Homer | 1e-4 | -1.110e+01 | 0.0002 | 3698.0 | 21.54% | 6603.2 | 20.25% | motif file (matrix) | svg |
| 40 | C T G A T C A G A G T C C G T A A T C G A T G C C G A T A C T G A G T C G A C T A T C G A G T C | MyoD(bHLH)/Myotube-MyoD-ChIP-Seq(GSE21614)/Homer | 1e-4 | -1.069e+01 | 0.0002 | 2890.0 | 16.83% | 5116.4 | 15.69% | motif file (matrix) | svg |
| 41 | C G A T T A C G T G A C G A C T C A T G C G T A T A C G A C G T G T A C C T G A | Bach2(bZIP)/OCILy7-Bach2-ChIP-Seq(GSE44420)/Homer | 1e-4 | -1.034e+01 | 0.0003 | 283.0 | 1.65% | 421.0 | 1.29% | motif file (matrix) | svg |
| 42 | C T G A C A G T C T G A A G T C C T A G G A C T A T C G G T A C | HIF-1b(HLH)/T47D-HIF1b-ChIP-Seq(GSE59937)/Homer | 1e-4 | -1.019e+01 | 0.0004 | 3834.0 | 22.33% | 6877.1 | 21.09% | motif file (matrix) | svg |
| 43 | T A C G T A C G G T A C A T C G T A C G T A C G G T C A C T G A C G T A G A C T | E2F4(E2F)/K562-E2F4-ChIP-Seq(GSE31477)/Homer | 1e-4 | -1.014e+01 | 0.0004 | 2478.0 | 14.43% | 4368.2 | 13.40% | motif file (matrix) | svg |
| 44 | T G A C A G T C C T G A T G A C C G T A A C G T A C G T A G T C A G T C C G T A | TEAD1(TEAD)/HepG2-TEAD1-ChIP-Seq(Encode)/Homer | 1e-4 | -9.407e+00 | 0.0008 | 1397.0 | 8.14% | 2404.3 | 7.37% | motif file (matrix) | svg |
| 45 | A T G C G T A C C G T A A G C T G C A T T A C G A G C T A G C T A G T C A G C T | Sox6(HMG)/Myotubes-Sox6-ChIP-Seq(GSE32627)/Homer | 1e-3 | -8.731e+00 | 0.0016 | 2872.0 | 16.73% | 5126.5 | 15.72% | motif file (matrix) | svg |
| 46 | C A G T G A C T C G T A G C T A G T A C G A T C G T A C G A C T A G C T A C G T T G A C C G T A C A G T A C G T A T G C | ZNF652/HepG2-ZNF652.Flag-ChIP-Seq(Encode)/Homer | 1e-3 | -8.246e+00 | 0.0025 | 424.0 | 2.47% | 678.6 | 2.08% | motif file (matrix) | svg |
| 47 | C T A G A C T G T G C A G T C A A T G C C G T A A T C G A T G C A G T C C T A G | ZNF341(Zf)/EBV-ZNF341-ChIP-Seq(GSE113194)/Homer | 1e-3 | -8.176e+00 | 0.0026 | 2670.0 | 15.55% | 4765.5 | 14.61% | motif file (matrix) | svg |
| 48 | T A G C T A G C G C A T C A T G A C T G G C T A C G T A A C G T A C T G G A T C | TEAD4(TEA)/Tropoblast-Tead4-ChIP-Seq(GSE37350)/Homer | 1e-3 | -8.089e+00 | 0.0028 | 1231.0 | 7.17% | 2124.2 | 6.51% | motif file (matrix) | svg |
| 49 | A G C T C T A G T G A C C G T A A C G T C G A T A G T C A G T C C T G A C A T G | TEAD3(TEA)/HepG2-TEAD3-ChIP-Seq(Encode)/Homer | 1e-3 | -8.058e+00 | 0.0028 | 1631.0 | 9.50% | 2854.8 | 8.75% | motif file (matrix) | svg |
| 50 | A G T C C G A T C T G A C G T A A C G T C A G T T C A G T G A C | Isl1(Homeobox)/Neuron-Isl1-ChIP-Seq(GSE31456)/Homer | 1e-3 | -7.893e+00 | 0.0033 | 2998.0 | 17.46% | 5380.4 | 16.50% | motif file (matrix) | svg |
| 51 | G A C T C G A T C T G A G T C A G A C T C G A T T C G A C G T A G C T A G C T A T G A C G T A C C G T A A C T G T G C A C G A T A C T G A C G T | Pitx1:Ebox(Homeobox,bHLH)/Hindlimb-Pitx1-ChIP-Seq(GSE41591)/Homer | 1e-3 | -7.683e+00 | 0.0040 | 336.0 | 1.96% | 530.0 | 1.63% | motif file (matrix) | svg |
| 52 | C G A T C T G A C G T A C A G T A G T C G A T C G A T C A C T G | Pitx1(Homeobox)/Chicken-Pitx1-ChIP-Seq(GSE38910)/Homer | 1e-3 | -7.479e+00 | 0.0048 | 6591.0 | 38.39% | 12126.1 | 37.19% | motif file (matrix) | svg |
| 53 | A C T G G A T C G A C T A C T G A C G T C A T G A C T G A C G T A G C T C G A T | RUNX-AML(Runt)/CD4+-PolII-ChIP-Seq(Barski\_et\_al.)/Homer | 1e-3 | -7.005e+00 | 0.0075 | 1178.0 | 6.86% | 2046.1 | 6.27% | motif file (matrix) | svg |
| 54 | T C G A C A T G C A T G T A C G G T A C T C G A A G T C C A G T C T G A C G T A A G T C G A C T | ZNF264(Zf)/HEK293-ZNF264.GFP-ChIP-Seq(GSE58341)/Homer | 1e-3 | -6.961e+00 | 0.0077 | 1574.0 | 9.17% | 2771.7 | 8.50% | motif file (matrix) | svg |
| 55 | G C A T A T C G C A T G G T A C G C T A A G T C T C A G T G A C G T C A T G C A | Arnt:Ahr(bHLH)/MCF7-Arnt-ChIP-Seq(Lo\_et\_al.)/Homer | 1e-2 | -6.784e+00 | 0.0091 | 2324.0 | 13.54% | 4158.9 | 12.75% | motif file (matrix) | svg |
| 56 | A C T G C A G T A C G T C G T A C G T A A C G T A C T G C T G A | Nkx6.1(Homeobox)/Islet-Nkx6.1-ChIP-Seq(GSE40975)/Homer | 1e-2 | -6.647e+00 | 0.0102 | 3979.0 | 23.18% | 7244.3 | 22.22% | motif file (matrix) | svg |
| 57 | G A C T C T A G G A T C C A G T A C T G C T G A A T G C G C A T A T G C C T G A | MafA(bZIP)/Islet-MafA-ChIP-Seq(GSE30298)/Homer | 1e-2 | -6.375e+00 | 0.0131 | 1796.0 | 10.46% | 3192.7 | 9.79% | motif file (matrix) | svg |
| 58 | T C G A A G C T A C G T A C G T A G T C A G T C A C G T A T C G G A C T A T C G | EWS:ERG-fusion(ETS)/CADO\_ES1-EWS:ERG-ChIP-Seq(SRA014231)/Homer | 1e-2 | -5.916e+00 | 0.0205 | 1120.0 | 6.52% | 1960.4 | 6.01% | motif file (matrix) | svg |
| 59 | A C T G C G T A A C T G A T G C T G A C G A T C A T C G T G C A A C T G A G T C | ZNF519(Zf)/HEK293-ZNF519.GFP-ChIP-Seq(GSE58341)/Homer | 1e-2 | -5.888e+00 | 0.0207 | 1164.0 | 6.78% | 2041.1 | 6.26% | motif file (matrix) | svg |
| 60 | A G T C G T A C A G C T C T A G A G T C C G A T A C T G C G T A A C T G G T C A | Zic(Zf)/Cerebellum-ZIC1.2-ChIP-Seq(GSE60731)/Homer | 1e-2 | -5.887e+00 | 0.0207 | 2888.0 | 16.82% | 5230.9 | 16.04% | motif file (matrix) | svg |
| 61 | G C T A C T G A T C G A A G T C A G T C C T G A A G T C G T C A C T G A T G C A | RUNX1(Runt)/Jurkat-RUNX1-ChIP-Seq(GSE29180)/Homer | 1e-2 | -5.805e+00 | 0.0217 | 1617.0 | 9.42% | 2875.6 | 8.82% | motif file (matrix) | svg |
| 62 | C A T G A C G T A G T C G A T C G A T C G A T C G C T A C T A G C T A G C T A G T C A G T C G A | EBF1(EBF)/Near-E2A-ChIP-Seq(GSE21512)/Homer | 1e-2 | -5.798e+00 | 0.0217 | 2646.0 | 15.41% | 4782.4 | 14.67% | motif file (matrix) | svg |
| 63 | A C G T C T A G A G C T A C G T A C G T C T G A A G T C G A C T A G C T C G T A | FOXM1(Forkhead)/MCF7-FOXM1-ChIP-Seq(GSE72977)/Homer | 1e-2 | -5.377e+00 | 0.0323 | 1676.0 | 9.76% | 2994.7 | 9.18% | motif file (matrix) | svg |
| 64 | A G T C C T G A A G T C C G A T C A G T G A T C A T G C A C T G A T C G G A C T | Fli1(ETS)/CD8-FLI-ChIP-Seq(GSE20898)/Homer | 1e-2 | -5.307e+00 | 0.0341 | 2664.0 | 15.52% | 4830.4 | 14.81% | motif file (matrix) | svg |
| 65 | G C T A T C G A C G T A C T A G A G C T G T C A G T C A C G T A A G T C C G T A | FOXA1(Forkhead)/MCF7-FOXA1-ChIP-Seq(GSE26831)/Homer | 1e-2 | -5.177e+00 | 0.0382 | 1636.0 | 9.53% | 2925.7 | 8.97% | motif file (matrix) | svg |
| 66 | T C A G T A C G G A T C G T A C T C G A C G A T C T G A C G T A G C T A C G T A | Hoxd11(Homeobox)/ChickenMSG-Hoxd11.Flag-ChIP-Seq(GSE86088)/Homer | 1e-2 | -5.164e+00 | 0.0382 | 4066.0 | 23.68% | 7457.5 | 22.87% | motif file (matrix) | svg |
| 67 | T A G C C G A T T A C G A C T G A G T C A C T G A T C G A T C G C G T A C T G A | E2F1(E2F)/Hela-E2F1-ChIP-Seq(GSE22478)/Homer | 1e-2 | -5.156e+00 | 0.0382 | 1613.0 | 9.40% | 2883.4 | 8.84% | motif file (matrix) | svg |
| 68 | C A G T T C A G G A T C A C T G A C G T C T A G A C T G A C T G G A C T C T A G | Egr1(Zf)/K562-Egr1-ChIP-Seq(GSE32465)/Homer | 1e-2 | -5.120e+00 | 0.0387 | 2870.0 | 16.72% | 5220.3 | 16.01% | motif file (matrix) | svg |
| 69 | A G T C G A C T A C T G G A T C G T A C C G T A T G A C A G T C C G A T A G C T A C G T A C G T C T A G G A C T C T G A | ZNF7(Zf)/HepG2-ZNF7.Flag-ChIP-Seq(Encode)/Homer | 1e-2 | -5.112e+00 | 0.0387 | 901.0 | 5.25% | 1575.9 | 4.83% | motif file (matrix) | svg |
| 70 | A T G C T A C G A G C T T G C A C G T A C G A T A C G T C T G A | DLX5(Homeobox)/BasalGanglia-Dlx5-ChIP-seq(GSE124936)/Homer | 1e-2 | -4.837e+00 | 0.0498 | 1338.0 | 7.79% | 2383.1 | 7.31% | motif file (matrix) | svg |
| 71 | C G T A T A G C T A G C T G C A A C T G C T A G C G T A C G T A T C A G G A C T | EHF(ETS)/LoVo-EHF-ChIP-Seq(GSE49402)/Homer | 1e-2 | -4.787e+00 | 0.0516 | 2371.0 | 13.81% | 4300.0 | 13.19% | motif file (matrix) | svg |
| 72 | T C A G C T A G C T A G C T A G T C A G T C G A C T G A C G A T A G T C G A T C A G T C T G A C | NFkB-p50,p52(RHD)/Monocyte-p50-ChIP-Chip(Schreiber\_et\_al.)/Homer | 1e-2 | -4.710e+00 | 0.0551 | 628.0 | 3.66% | 1085.8 | 3.33% | motif file (matrix) | svg |
| 73 | G A C T C T A G C T A G A G T C T G C A A C T G A C G T A C G T C T A G T C A G | AMYB(HTH)/Testes-AMYB-ChIP-Seq(GSE44588)/Homer | 1e-2 | -4.661e+00 | 0.0570 | 3485.0 | 20.30% | 6386.3 | 19.58% | motif file (matrix) | svg |
